# An Ultrasound Tomography Method for Monitoring CO_2_ Capture Process Involving Stirring and CaCO_3_ Precipitation

**DOI:** 10.3390/s21216995

**Published:** 2021-10-21

**Authors:** Panagiotis Koulountzios, Soheil Aghajanian, Tomasz Rymarczyk, Tuomas Koiranen, Manuchehr Soleimani

**Affiliations:** 1 Engineering Tomography Laboratory (ETL), Department of Electronic and Electrical Engineering, University of Bath, Bath BA2 7AY, UK; p.koulountzios@bath.ac.uk; 2 Research & Development Centre Netrix S.A., Wojciechowska 31, 20-704 Lublin, Poland; soheil.aghajanian@lut.fi (S.A.); tomasz@rymarczyk.com (T.R.); 3 School of Engineering Science, LUT University, Yliopistonkatu 34, 53850 Lappeenranta, Finland; tuomas.koiranen@lut.fi

**Keywords:** ultrasound computed tomography, travel-time imaging, time-of-flight transmission imaging, mixing process, crystallization process, CO_2_ capturing via crystallization

## Abstract

In this work, an ultrasound computed tomography (USCT) system was employed to investigate the fast-kinetic reactive crystallization process of calcium carbonate. USCT measurements and reconstruction provided key insights into the bulk particle distribution inside the stirred tank reactor and could be used to estimate the settling rate and settling time of the particles. To establish the utility of the USCT system for dynamical crystallization processes, first, the experimental imaging tasks were carried out with the stirred solid beads, as well as the feeding and stirring of the CaCO_3_ crystals. The feeding region, the mixing process, and the particles settling time could be detected from USCT data. Reactive crystallization experiments for CO_2_ capture were then conducted. Moreover, there was further potential for quantitative characterization of the suspension density in this process. USCT-based reconstructions were investigated for several experimental scenarios and operating conditions. This study demonstrates a real-time monitoring and fault detection application of USCT for reactive crystallization processes. As a robust noninvasive and nonintrusive tool, real-time signal analysis and reconstruction can be beneficial in the development of monitoring and control systems with real-world applications for crystallization processes. A diverse range of experimental studies shown here demonstrate the versatility of the USCT system in process application, hoping to unlock the commercial and industrial utility of the USCT devices.

## 1. Introduction

Industrial Process Tomography (IPT) offers great prospects to visualize the state of vessels, pipes, and tanks containing multi-phase flows and mixtures without being destructive and invasive to the process. Nowadays, there is a great need for monitoring the internal functions of the processes to further improve the design and operation. Therefore, the application of IPT in a wide variety of industrial applications is thoroughly being researched, such as fluid transport in pipes, and the manufacturing of paint, detergents, food, cosmetics, and pharmaceutical operations.

Industrial reactors are broadly used in different unit operations, making the tomographic studies on their functionality a hot topic for the research communities [1,2,3,4,5,6,7]. Slurry transportation processes can also be found in many process reactors, for example, dredging, food processing, and nuclear waste management. Slurry flow usually involves moving high-density material through a carrier liquid (e.g., water), with few tomographic studies being focused on slurry mixtures [8,9,10,11]. Mixing in industrial reactors is critical and challenging, especially in the case of complex fluid rheology. There are different types of mixing processes: liquid–liquid mixing, gas–liquid mixing, solid–liquid mixing, and the mixing of multiphase non-Newtonian fluids. Furthermore, the challenges can be further increased when the product has high-quality requirements for the degree of homogenization, and the end-product has limited tolerance to variations in the hydrodynamics conditions in the reactor. As the mixing phenomena can significantly affect the quality of the end products, it is of significant importance to utilize monitoring instrumentation for detailed analysis and to perform an efficient operation in terms of final yield.

Crystallization is a key reaction in many pharmaceutical/chemical sectors. Among several types of crystallization processes, the industrial demand for reactive crystallization (also known as precipitation) has been increasing in recent years [12]. The growth is mostly due to the energy efficiency of these processes in comparison to, for instance, cooling or evaporative crystallization. In reactive crystallization, the formation of solid particles from solution is very fast and reactions are instantaneous, which causes local variations in density and supersaturation gradient. In these processes, efficient mixing at different scales (i.e., micro-, meso-, and macro-mixing) becomes a critical factor and has a significant impact on process characteristics such as crystal size distribution and shape [13]. Coupling the fast reaction kinetics with the complex nature of the fluid flow hydrodynamics renders the monitoring and control of the unit operations challenging. The real-time characterization and control of fast kinetic crystallization systems have profound importance in ensuring end-product quality in these mixing sensitive processes. The main control parameters have been the subjects of intensive research. Normally, yield and quality targets for batch crystallization are given in terms of solute concentration and crystal size distribution (CSD). Several multipurpose and reliable methods measuring these characteristics have been studied, which are mainly based on the use of in situ spectroscopic techniques. Such techniques include mid- and near-infrared, Raman, UV-visible, and ultrasound spectroscopy [14,15,16,17,18]. Regarding ultrasound techniques, nonspectroscopic methods have been studied in the form of single-frequency acoustic emission with promising results, leading to less complex measurement setups [19,20]. Moreover, the influence of mixing speed on crystal size distribution (CSD) has been studied widely [21,22,23]. However, due to the abovementioned complexities, the development of functional monitoring and control schemes for these fast kinetic processes is still an ongoing research topic [24].

Contrary to the abovementioned crystallization monitoring single-point measurement methods, there has not yet been any applicability of tomographic methods for the control of such processes. Tomography could add useful information regarding the topology and the material phase distributions in mixtures. It can also offer the opportunity to quantify the degree of homogeneity of particulate suspensions and other multiphase mixtures. Even though tomographic techniques cannot compete against the spectroscopic methods in PSD measurements, they can add useful spatial information and can be used as a fault detection or quality assurance tool for the complex mixing processes. They can also be used as complementary alternative measurements to the PSD single-point measurements aiming at multiple measurements integration. Few tomographic studies have been conducted for monitoring in situ crystallization processes such as X-ray microtomography [25,26], X-ray diffraction tomography [27], electrical resistance tomography [28,29,30], electrical capacitance tomography (ECT) [31], and ultrasound tomography [32]. The reconstructed tomograms can help to identify the reaction endpoint and ensure the chemical reactions are controlled precisely. In reactor processes—particularly in crystallization—integrated tomographic visualization, data fusion, and machine learning have the potential to be utilized as a complementary method to the conventional point-based measurement techniques, aiming at fault detection, suspension density and spatial distributions characterization, and PSD indications. The USCT can be used in a contactless fashion as it was in this study, offering an advantage over EIT that requires direct contact with the medium. Furthermore, the sensitivity of EIT is reduced in the central area of the reactor tank, while USCT can offer a good and uniform resolution over all regions. More commercial availability of the EIT devices could be a reason for their use in various processes. This work aimed to demonstrate that the USCT is a very versatile IPT tool and deserves further commercial development for deployment in real-life applications.

In the present work, an ultrasound computed tomography system was utilized to investigate the reactive crystallization process of calcium carbonate. USCT offers some major advantages over other tomographic methods, offering a low-cost alternative that could reach suitable outcomes in spatial and temporal resolution. The calcium carbonate production is integrated with a carbon capture process where ${\mathrm{CO}}_{2}$ gas is absorbed into sodium hydroxide solutions. The idea presents real-world applicability for carbon dioxide utilization and valorization to an economically attractive chemical [33]. The utilized USCT measurement system is based on acoustic impedance features to characterize and image acoustical distributions. USCT measurements and reconstruction provided insights into the bulk particle distribution inside the stirred reactor and were used to estimate the settling velocity of the particles. The study gave insights into the crystallization system and aimed to link the online tomographic measurements to fault detection and malfunction identification when out-of-specification events occur throughout the process. The paper demonstrated an extensive range of uses for a versatile USCT system.

It is worth noticing that the USCT technology has reached remarkable image resolution that is comparable to MRI and XCT for various medical applications, as well as geophysical applications [34,35,36]. The medical applications of the USCT for brain and breast imaging can be considered as static-type imaging, so a very large number of (smaller size) transducers could be used. The same could be carried out in industrial process applications. However, the limiting factor is the dynamical nature of most of the process monitoring applications. Deployment of a very large number of sensors leads to extensive measurement time and extensive computational time.

## 2. USCT System and Method

**Figure 1:**
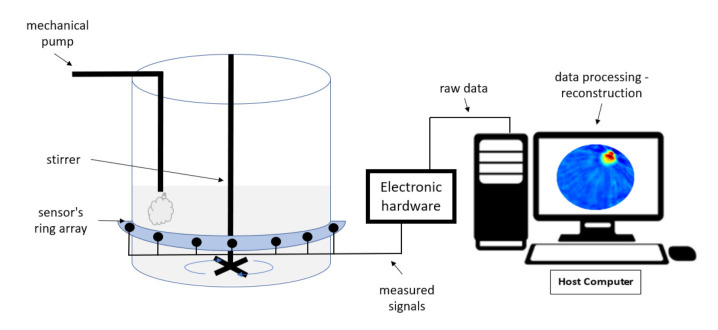
A tank reactor with the integrated USCT system.

The USCT system in this study is based on transmission-mode USCT data. Recorded waveforms are used to reconstruct travel-time delays and acoustic attenuation (AA) profiles using the time-of-flight (TOF) of the first arrival pulse and its amplitude, respectively. Figure 1 depicts the tomographic setup focused on a tank reactor. The tomographic setup consists of the sensor’s ring array, the electronic hardware/controller, and the host computer, which is responsible for the tomographic software and displaying the results. The reactor tank is made of acrylic material and is equipped with a feeding pump and a stirrer. The sensor’s array is defined by 16 piezoelectric transducers. They work as both transmitter and receiver, mounted at the outer boundary of the tank, using an ultrasonic glue. The instrument can provide TOF and amplitude data as it filters the full-waveform signal and shares the data with the host computer. For this pre-processing step, the electronic hardware is responsible as the filtering occurs in analog form. Therefore, the system’s temporal resolution is optimized, as a significant amount of data are cut off before the data transfer stage. The tomographic instrument measures the time needed for a wave to overcome the medium but also the energy that is being lost by absorption or back-scattering.

### 2.1. The USCT Hardware 

The USCT hardware system consists of a circular ring of 16 transducers, an electronic hardware setup for emitting and recording TOF and AA data of the first arrival pulse, and computer software for analysis and reconstructions. Each recording comprises 256 measurements, accounting for 16 recordings for every one of the 16 emissions that take place. The system provides 5 frames of computed TOF/AA values each second, hence resulting in an overall temporal resolution of 4 frames per second (fps). Such a response over time was used for tomographic reconstruction execution in the present work and is considered adequate for a wide range of dynamical processes, such as pipe-flow monitoring. The USCT system’s design features are presented in Table 1.

The system consists of eight four-channel measurement cards connected by an FD CAN bus with a measuring module, which is a bridge between the microprocessor measuring system and the panel. The measuring module monitors the course of the measurement, stores parameters, controls the high-voltage converter, and switches the bus. The four-channel measurement card was made in a modular manner. The sections of amplifying and filtering analog signals were made on small separate PCBs. For better isolation of individual channels, it is possible to mount a shielding housing on each module. In addition, a four-channel high-rectangular-voltage generator was designed at the bottom of the plate. The generator circuit consists of four four-channel MOSFET drivers connected to double H bridges (TC8220). A single section can generate a three-stage square wave signal (Vpp–GND–Vnn). Depending on the power source, the circuit can generate voltages from +100 V to −100 V. In the presented prototype, the generator circuit is powered by a symmetrical voltage of +/−24 V. Therefore, the maximum instantaneous current efficiency of MOSFET keys is 3 A. The converters were synchronized with a built-in counter in the STM32G474 microcontroller. The sampling data travel to the internal memory buffer by DMA. The generation of appropriate waveforms controlling the MOSFET keys of the four-channel high-voltage generator was carried out by cascading three meters. The microcontroller generates 4 square waveforms (for a positive key, for a negative key, and for two keys connected to the device’s ground). The control signals travel directly to all four generator sections. Each section has an input that activates or deactivates a given section. Thanks to that, it is not necessary to generate control signals separately for each section. The analog module is a system that amplifies the ultrasonic transmitting signal. The proposed design has an integrated AD8331 amplifier with gain control using an external DAC converter and a system converting the AC signal to ADL5511 envelopes and two THS4521 differential amplifiers. Due to the distance between the modules and the microcontroller and the presence of a high-voltage generator on the same PCB, the final output signal from the module is a symmetrical differential signal, which reduces the amount of noise.

**Figure 2:**
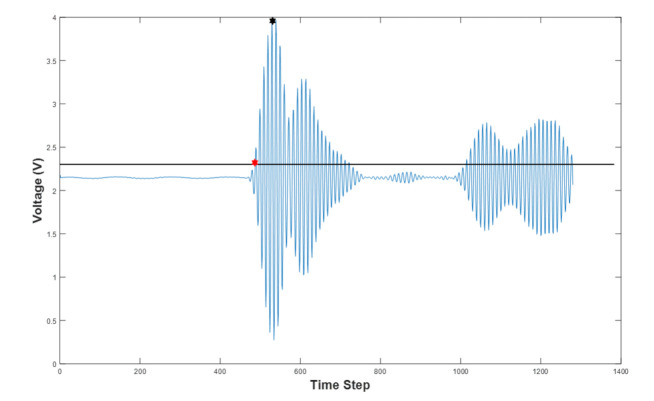
Recorded full-waveform signal from 1st transmitter-6th receiver pair.

Figure 2 shows the full waveform signal used for the selection of TOF and AA values. A minimum threshold of 10% was used to cut down the minor pulses caused by back-scattering or equipment-related noise. The threshold’s level is presented in Figure 2 by the black horizontal line. The TOF value is determined by the projection of the first signal’s point after the threshold to the *x*-axis (time step axis), shown by the red point in the corresponding graph. The biggest y-value (the *y*-axis is a measurement value that represents the amplitude of the receiving wave) within a 20% signal’s window in the transmitted pulse “region” indicates the recorded pulse’s amplitude, depicted by the black point in the graph. A more detailed method for signal picking can be seen in [37], where the hardware is also briefly described. In both TOF and amplitude data, the “Deleting Outliers” statistical filtering method was used to handle the noise coming from multi-backscattering. An iterative implementation of the Grubbs Test, which checks one value at a time, was used to identify the outlier signals [38]. The MCUSD11A400B11RS transducer (ultrasonic sensor, frequency 400 kHz +/−16 kHz, diameter 11 mm, material made of aluminum, input voltage 300 Vp-p, directivity (−3 dB) 7° +/− 2°, operating temperature −20 °C to 80 °C. Manufacturer: MULTICOMP) was used for the sensor array. UST1.0 with 40 kHz operation was used in [39,40].

### 2.2. Image Reconstruction 

The tomographic approach uses the transmission sensitivity matrix that simulates the propagation of the measured energy from sensors. The measurement data for transmission tomography include *TOF* data and Amplitude Attenuation (AA) data. *TOF* measurement data come from the subtraction of background data from the full data, and they define the travel-time delays in microseconds (μs).

(1) $$TOF=TOF_{full}-TOF_{back}$$

*AA* measurement data [41] are computed by

(2) $$AA=\frac{1}{f_{c}}\ln\left(\frac{AA_{full}}{AA_{back}}\right)$$

where $AA_{back}$ is the reference amplitude data and $AA_{full}$ is the data when the domain changes from background, namely the data that come during the process and describe the changes in the acoustic field. $f_{c}$ is the center frequency of the excitation pulse.

Each spherical wave can be approximated by a cone of rays and, subsequently, every plane wave by a fan of rays. Using the recorded *TOF* or *AA* values, one can calculate the average sound speed or acoustic attenuation for the path of the modeled ray. To generate the propagation model of the emitting energy, a computational model based on diffraction on the 1st Fresnel zone is utilized [42]. Fresnel volume or ‘fat ray’ tomography is an appealing compromise between the efficient ray theory tomography and the computationally intensive full waveform tomograph [43]. Using a finite frequency approximation to the wave equation leads to a sensitivity kernel where the sensitivity of the travel time delay also appears in a zone around the fastest ray path. The delay time is given as:

(3) $$\Delta t\left(x\right)=t\left(s,x\right)+t\left(x,r\right)-t_{0}\left(s,r\right)$$

where $t\left(s,x\right)$ and $t\left(x,r\right)$ are the travel times from the source(s) to the point x and from x to the receiver (*r*), respectively, and $t_{0}\left(s,\;r\right)$ is the travel time along the ray path from source to receiver. One can evaluate the times of traveling using the ray-tracing method. A point x is always within the first Fresnel zone if the corresponding travel time satisfies the following equation, in which T defines the emitted wave’s period:

(4) $$\left|\Delta t\left(x\right)\right|<\frac{T}{4}$$

The sensitivity of a Frechet kernel based on the first Fresnel zone is defined as follows:

(5) $$D\left(x\right)=K\;V\left(s,x\right)\;V\left(x,r\right)\;\cos\left(2\pi \frac{\Delta t\left(\chi \right)}{Τ}\right)\;exp\left(-{\left(\frac{a\Delta t\left(x\right)}{\frac{T}{4}}\right)}^{2}\right)$$

where *D*(*x*) is the sensitivity at domain’s point *x*, *V*(*s*,*x*) is the amplitude at *x* of the wavefield propagating from source s, *V*(*x*,*r*) is the amplitude at receiver point *r* with the wavefront emitted from point *x*, and *K* is the normalization constant. The cosine factor models the alternating sensitivity being positive in the odd Fresnel zones and negative in the even Fresnel zones. a denotes the Gaussian factor, which controls the degree of cancellation in Fresnel zones beyond the first. The SIPPI MATLAB software was used to generate the sensitivity kernels [44]. To develop the USCT system of the present work, a Frechet sensitivity map described by Buursnick et al. and produced by the SIPPI MATLAB package was employed [43]. The sensitivity matrix represents a linearly modeled distribution of acoustic propagation that can fit well with *TOF* and *AA*.

(6) $$A_{i,j}=\frac{D_{i,j}}{\sum_{i_{1}=1}^{m}\sum_{j_{1}=j}D_{i_{1},j_{1}}}$$

where $S_{i,j}$ is the sensitivity matrix based on the Frechet method and $A_{i,j}$ is the normalized matrix, which is used for reconstructions, with $i=\left[1,\ldots m\right]$ and $j=\left[1,\ldots ,n\right]$.

A generalized tomographic forward problem uses the approximated excitation’s sensitivity model and the known domain to recover the measurement data. In our approach, TOF or AA differences can be used as measurement data ΔM. It can be expressed as:

(7) ΔM= A ΔS+e

where ΔM is the measured data from sensors and ΔS is the reconstructed distribution based on acoustic features. A represents the modeling operator that expresses the sensitivity distribution in the FOV, and e is the noise in the measurements. A simplified inversion can be performed using linear back-projection (LBP):

(8) $$\Delta S\;\approx \;A^{T}\;\Delta M$$

Generally, the LBP leads to low-quality USCT imaging results. Advanced regularization-based methods can produce high-quality images. To solve the regularized inverse problem with a high degree of stability, the robust total variation (TV) regularization algorithm is applied [39]. The TV problem is defined as an optimization problem as follows:

(9) $$minA\left(\Delta S\right)={\left|\left|\left(A\;\Delta S+e\right)-\Delta M\right|\right|}^{2}+a\;{\left|\left|\nabla \Delta M\right|\right|}_{1}$$

where a is the regularization parameter, ∇ is the gradient, and ${\left|\left|.\right|\right|}_{1}$ is the $l_{1}$−norm. Subsequently, the problem to be solved is the constrained optimization problem, as shown in Equation (10) given a priori known noise level *p*:

(10) $$x_{a}=argmin{\;}_{\Delta S}\;\left(\;\alpha \;{\left|\left|\nabla \Delta S\right|\right|}_{1}\right)\;\mathrm{such}\;\mathrm{that}\;{\left|\left|A\;\Delta S-\Delta M\right|\right|}^{2}<p$$

The constrained optimization problem is solved by the Split Bregman-based TV algorithm [45]. To optimize the tomographic images, the negative effects of undesired artefacts are removed by careful selection of the regularization parameter.

## 3. CaCO_3_ Crystallization Setup and Process Description

Ultrasonic measurements have a proven efficiency in the characterization of suspension densities and slurry mixtures. Prior studies have shown the relation between phase velocity and acoustic attenuation on growing suspensions and different frequency excitations [46,47]. These studies were based on single measurements, providing an indication of the average changes in the entire domain. The USCT method can extend these to provide local and regional information. In crystallization cases, the regions of well-dispersed crystals and regions of associated crystal networks could exist together. Moreover, accounting for the vigorous stirring process and the aeration that could be introduced to the mixture, a three-phase flow would occur by a region of dispersed gas/solid/liquid phase (liquid solution-crystalline particles-bubbles). Acoustic attenuation can be reduced further as sound propagates through the dispersed phase, due to multiple back-scattering. Regarding time-of-flight, delays are noticeable in the propagating signals over frames due to the forming dispersed phase and the low-frequency excitation of 400 kHz.

The micron-sized crystallization process of the present work is part of the carbon capture and utilization scheme where process monitoring with the USCT system is presented. Carbon dioxide is scarcely soluble in water under the standard temperature and pressure conditions. ${\mathrm{CO}}_{2}$ absorption is improved by increasing the pH of water so that the resultant chemical reaction becomes very fast at higher pH values. Sodium hydroxide–water with pH 14.10 ± 0.1 was used to prepare the reagent solution for the crystallization process. A small-scale ${\mathrm{CO}}_{2}$ bottle (purity > 99.99%) was employed to demonstrate the carbon capture process and to inject the gas into the solutions. The operation was performed by first collecting the CO_2_-loaded solutions, and later using them for calcium carbonate crystallization. In the process under investigation, the semi-batch feed to the stirred tank reactor contained dissociated ${\mathrm{CO}}_{3_{\left(\mathrm{aq}\right)}}^{2-}$, ${\mathrm{OH}}_{\left(\mathrm{aq}\right)}^{-}$, and ${\mathrm{Na}}_{\left(\mathrm{aq}\right)}^{+}$ ionic solution at a pH range of 12 ± 0.1. The governing chemical reaction is presented in Equation (11) where aqueous ${\mathrm{CO}}_{3_{\left(\mathrm{aq}\right)}}^{2-}$ flows through an inlet pipe (diameter: 2 mm) into the crystallizer containing a known concentration of calcium chloride (${\mathrm{CaCl}}_{2}$, purity > 98%, Merck). A detaileds description of the CO_2_ capture process and its integration with the calcium carbonate crystallization is given in [48].

(11) $${\mathrm{CO}}_{3}^{2-}{}_{\left(\mathrm{aq}\right)}+2{\;\mathrm{Na}}^{+}{}_{\left(\mathrm{aq}\right)}+{\mathrm{CaCl}}_{2}{}_{\left(\mathrm{aq}\right)}\to {\mathrm{CaCO}}_{3}{}_{\left(s\right)}↓+2{\;\mathrm{NaCl}}_{\left(\mathrm{aq}\right)}$$

**Figure 3:**
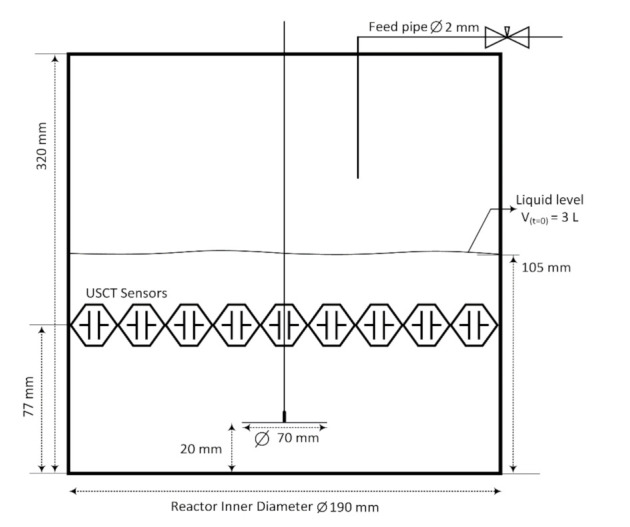
Schematics of the experimental setup. Dimensions of the plexiglass reactor and position of the USCT sensors. The initial solution volume in the tank is 3 L.

The feed addition rate to the receiving reactor was constant at 40 ${\mathrm{mL}\;\min}^{-1}$ during the whole experiment. All the experimental runs were carried out at a temperature of 20 ± 2 °C. Figure 3 shows the schematics of the utilized crystallization reactor.

**Figure 4:**
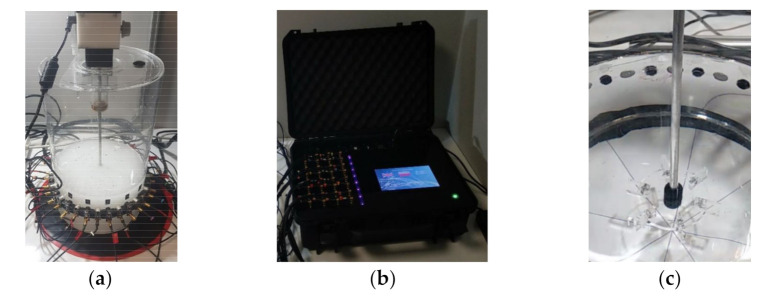
Ultrasound experimental setup. (**a**) Reactor tank, (**b**) measurement unit, (**c**) mixer.

Figure 4 shows photographs of the entire experimental setup in which the USCT system was utilized to conduct process monitoring. The crystallization reactor was made of plexiglass with an inner diameter of 190 mm, and a plastic-made, flat-blade Rushton impeller was used for agitation.

## 4. Results and Discussion

Several dynamical experiments were specifically designed to evaluate the USCT response to various process events. The effects of certain operator-induced malfunctions including switching on/off the stirrer and the feed pump, particle addition, and phase changes were investigated. A description of the experimental results is presented in the following sections. In the presented work, we show the potential applications of the USCT in chemical reactor processes and especially in batch crystallization. USCT characterizes the medium by measuring the time of flight and acoustic attenuation of the sound propagation. Ultrasound tomographic imaging offers promising industrial applications due to its low-cost value and the ability to be nonintrusive to the whole process even though it is considered invasive to the process. First, the experimental tests focused on particles’ concentration and dynamical status characterization in mixing scenarios. As mixing highly affects the dynamical state of the tanks, it introduces noise to the system, which disturbs the ultrasonic measurements. Noise is always apparent in the measurements when the dynamics of the tank abruptly change. The main challenge, regarding ultrasonic online measurements in mixing environments, is the elimination of the “stirring noise” and the use of the quantitative information, without the measured data being seriously distorted.

### 4.1. Particle Beads Detection by Ultrasound Tomography

To establish the dynamical imaging based on particle concentration, circular particle beads of 4 mm in diameter were poured into the stirred tank reactor containing 3 L of water. The idea of the proposed experiment was to investigate the effects of mixing and the real-time changes in the dynamical states of the reactor by using USCT. A description of the experimental procedures is presented in Table 2.

**Figure 5:**
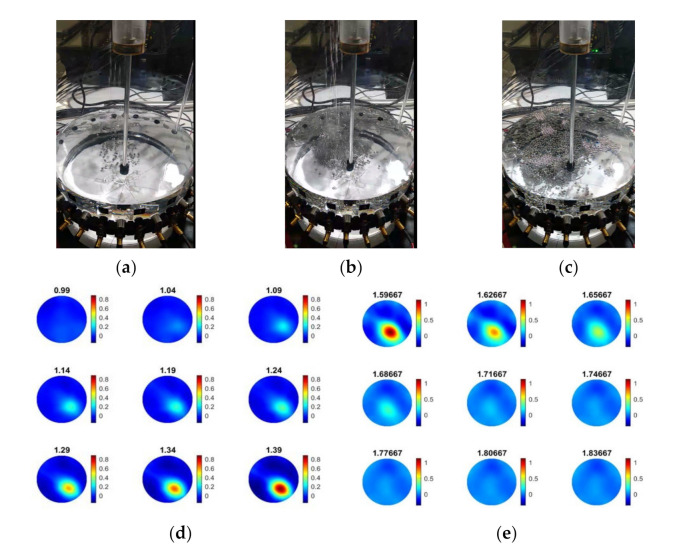
Addition of 100 g of particle beads in 3 L of water: (**a**–**c**) experimental photos demonstrating the pouring location and the mixing (200 RPM); (**d**) reconstructions between 0.99 min and 1.39 min, (**e**) reconstructions between 1.6 min and 1.84 min that corresponds to terminating the time of the addition of the particles and the settling period.

Figure 5 shows experimental photos and tomographic reconstructions over time during the experiment with 100 g of particle beads. Figure 5a–c present three distinguished states of the experiments during the first 2 min: (a) the beginning, (b) the middle, and (c) the end of particle addition. The sedimentation process of solid particle beads is clear in the presented experimental photographs. The 2D reconstructions show the gradually increasing values of the injection point as the particles were poured into the tank. Acoustic field inhomogeneities were introduced both due to the existence of particles within the sensors’ field-of-view (FOV) and the disturbances that occurred due to pouring. Figure 5d presents the reconstructed frames during the particles’ pouring, showing an increasing trend of TOF delays. Figure 5e displays the tank’s state immediately after the pouring and provides insights into the particle’s settling over time, as it is described by a decreasing trend of TOF delays.

**Figure 6:**
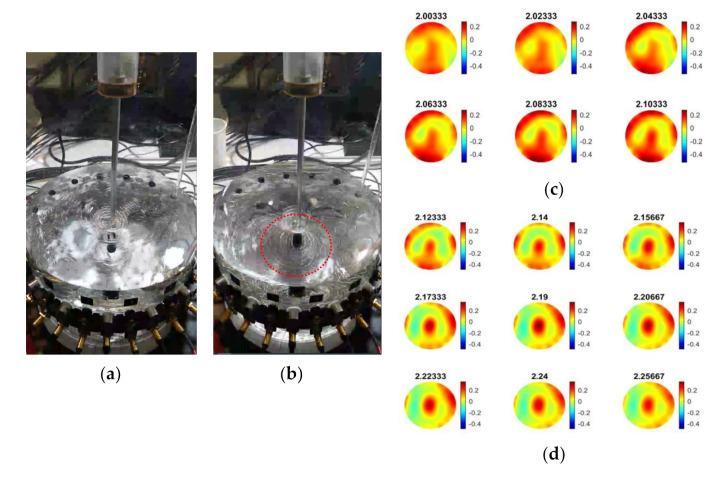
Monitoring the mixing process of 100 g of particle beads (diameter 4 mm) with USCT. (**a**,**b**): Experimental images of mixing at 200 RPM and the formation of a central vortex in the tank. (**c**,**d**): USCT-based tomographic reconstructions between 2 min and 2.25 min, showing the process of mixing. Amount of particles: 100 g, water volume: 3 L.

As tabulated in Table 2, mixing at 200 RPM was switched on at approximately T + 2 min into the experiment. Due to the mixing, the whole medium turned into a dispersed liquid—solid state, as displayed by the experimental photo in Figure 6a. Tomographic reconstruction addresses the mixing-induced inhomogeneities by compromising the higher intensity values across the entire region-of-interest (ROI), as shown in Figure 6c. According to Figure 6b, the particles tended to assemble in the tank’s central area (where the stirring took place), which is due to the central vortex formation and the centripetal force induced by the stirrer rotation. The reconstructions in Figure 6d were in good agreement with the experimental observations where higher inhomogeneities were formed at the central location after the start of the stirrer.

**Figure 7:**
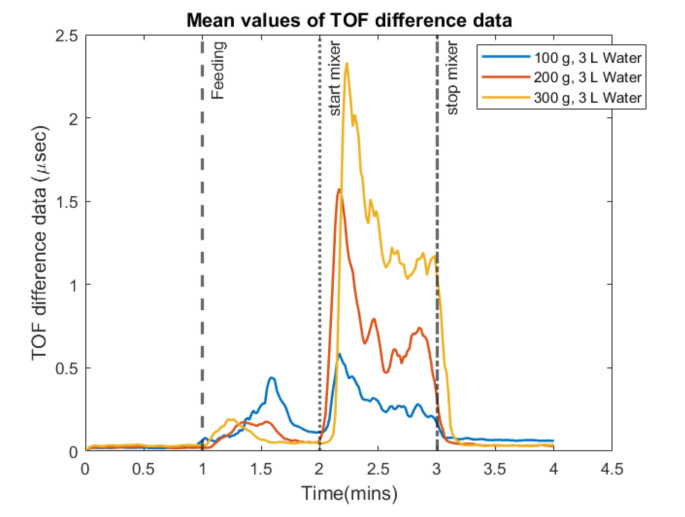
Mean values of the time-of-flight delay for the three experiments with different bead concentrations. The volume of water inside the reactor is constant at 3 L and 3 different amounts of particles are tested.

The exact experimental procedures as discussed above (Section 4.1) were conducted two more times, each time adding 100 g of additional particle beads to 3 L of water and recording the measurements. Figure 7 shows the dynamical analysis of the experiment using the mean value of the 256 measurements. All the three curves are described by a small prior bump that corresponds to the pouring and the disturbances that are caused and a latter one that corresponds to the concentration of the mixture, as it comes after the stirring starts. Comparing the three curves, quantification can be achieved with TOF imaging as the delays are related to the concentration of the two/phase mixture. For these experimental cases, AA data were analyzed, offering the same responses as the TOF data.

The experiment with the plastic beads offers insights into the system’s functionality in high-dynamical scenarios. First, efficiency was proven in detecting malfunction cases coming from localized higher concentrations and generated disturbances in the medium (by pouring). Then, quantification was achieved in different concentration tests. Finally, the specific flat blade turbine impeller introduced the specific pattern of gathering the plastic beads close to the stirring area (tank’s center).

### 4.2. Characterizing CaCO_3_ Solid Particles Distribution by USCT

**Figure 8:**
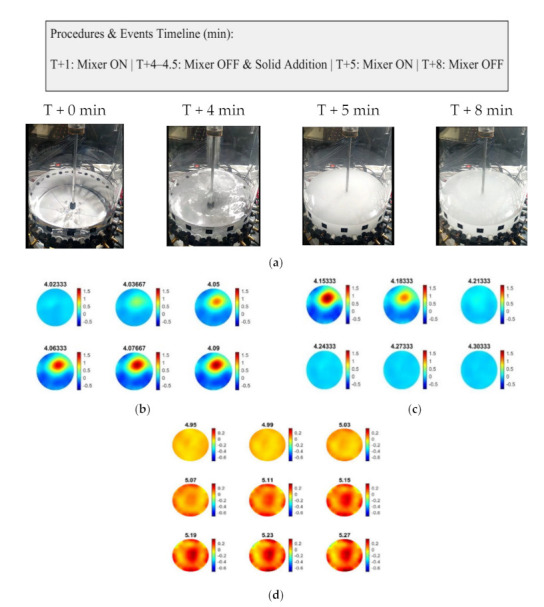
(**a**) Images on shown time step images of feeding. (**b**,**c**) Reconstruction between times 4.02 min and 4.30 min, describing feeding. (**d**) Reconstruction between times 4.95 min and 7.43 min during the mixing process.

Four different concentrations of solid calcium carbonate suspensions were used to investigate the sound propagation. Samples of commercial calcite (provided by VWR, purity > 99%) were added by hand from the top of the reactor containing 3 L of water. Figure 8a presents the experimental procedure in sequence. Figure 8b,c display the reconstructions over the pouring process of the calcium carbonate particles, while Figure 8d shows the reconstruction over the mixing process and provides insights into the mixture’s homogeneity. The tomographic images were obtained for the concentration of 25 ${g\;L}^{-1}$ at several key events and show the particle addition and also the effect of mixing that leads the medium to a homogeneous state.

**Figure 9:**
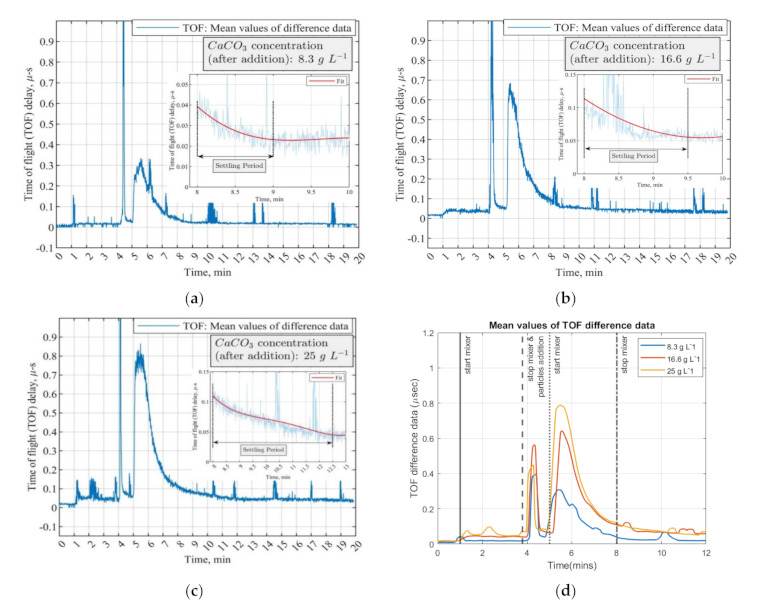
Mean values of the time-of-flight difference data in four concentrations of CaCO_3_ suspensions. Experimental procedures are the same for all the concentrations. The initial volume of water is 3 L. Settling period is defined to calculate the settling velocity of particles. (**a**–**c**) Plot for 8.3 g, 16.6 g, and 25 g; (**d**) all the mean values from (**a**–**c**) in one plot.

As presented in Figure 9, all the conducted experiments’ mean TOF data are defined in a similar graph. In T + 1 min, stirring starts in the reference medium (water) having almost zero impact on the mean TOF data. The highest peak at T + 4 min in the mean values graph is due to an abrupt change and disturbance in the reactor caused by the pouring of CaCO_3_ particles. Switching on the mixer at T + 5 min distributes the micron-sized particles in the suspension and causes a rapid increase in the TOF. The increase in the TOF delays agrees with the concentration increase. The TOF has a descending trend after reaching the maximum peak (ca. from T + 5.5 min afterward), which characterizes a relatively homogenous medium that facilitates sound propagation, concluding in the sound speed propagation decrease at higher concentrations of solid calcium carbonate in the reactor, resulting in higher TOF delays. For the corresponding experiments, AA data were analyzed, offering the same responses as the TOF data.

#### Quantification of Particle Settling Time by USCT Measurement

The settling velocity of particles is a function of the free settling velocity (terminal velocity): it decreases as the solid particle concentration increases in the fluid domain. The free settling velocity of particles for the Stokes’ regime, $V_{t}$*,* is determined based on the following expression:

(12) $$V_{t}=\frac{gd_{p}^{2}\left(\rho_{\sigma }-\rho_{f}\right)}{18\mu }$$

where $\rho_{f}$ and $\rho_{s}$ are fluid and solid density, respectively, $d_{p}$ represents particle diameter (mean particle diameter: 25 μ m), *g* is the gravitational acceleration (9.81 ${m\;s}^{-2}$), and μ denotes the dynamic viscosity of the fluid (0.0089 Pa s for water). The value of the free settling velocity is strongly dependent on higher volumetric concentrations of solids (φ); when a cloud of solid particles is settling in a quiescent liquid, additional interactions and hindering effects (i.e., increased drag caused by the proximity of particles) influence its settling velocity. A typical semiempirical approach known as Richardson–Zaki is based on the power-law function of volume fraction:

(13) $$V_{HS}=V_{t}{\left(1-\varphi \right)}^{n}$$

where $V_{HS}$ denotes the hindered settling velocity (i.e., corrected velocity), and n is a function of the particle Reynolds number and dilution degree of the suspension: *n* = 6.5 for $Re_{p}<0.2$. 

In the present work, the settling period was approximated based on the ultrasound tomographic measurements of the most diluted suspension (i.e., 8.3 ${g\;L}^{-1}$ ${\mathrm{CaCO}}_{3}$ concentration). The settling period is defined as the time interval for a cloud of solid particles to reach from the suspension surface to the plane of the sensors after the mixer is switched off—the distance from the suspension surface to the plane of the sensor is ca. 33 mm. A plateau in the measured mean value of the sound speed is obtained after the stirrer is switched off, as shown in Figure 9. The initial point of the plateau in the measurement is attributed to the time that solid particles are passing the plane of sensors.

As calculated based on Equations (12) and (13) for the abovementioned particle size, the average settling period is approximately 1 min for the solid volumetric concentration of 0.31% (8.3 ${g\;L}^{-1}$). The calculated value of 1 min is in good agreement with the estimated settling period from the USCT measurement (Figure 9a). However, in denser particle suspensions, the estimated settling periods based on USCT are higher up to 5 min for the case of 33.3 ${g\;L}^{-1}$ solids (φ=1.22%). According to Equation (13), the particle hindered settling velocity does not explain the estimated settling times by the USCT. Apparently, the denser suspensions of size-distributed particles tend to discharge from the measurement zone (plane of sensors). Moreover, considering that the tank is without any baffles, the fluid motion continues in the tangential direction after stopping the mixer. The effects of particle motion, travel path of particles, and settling times can be further investigated by Computational Fluid Dynamics (CFD) simulations and the Lagrangian particle tracking method alongside the USCT experiments.

### 4.3. Reactive Crystallization Monitoring by USCT

Ultrasound-based tomographic measurement was used to detect localized crystalline suspensions and monitor the reactive crystallization of the calcium carbonate process according to the chemical reaction in Equation (11). In all the cases, the initial concentration of calcium chloride was 1.6 $g\;L^{-1}$. The operating parameters of the experiments are listed in Table 3. The entire experimental procedure was repeated two times to ensure the repeatability of the measurements.

**Figure 10:**
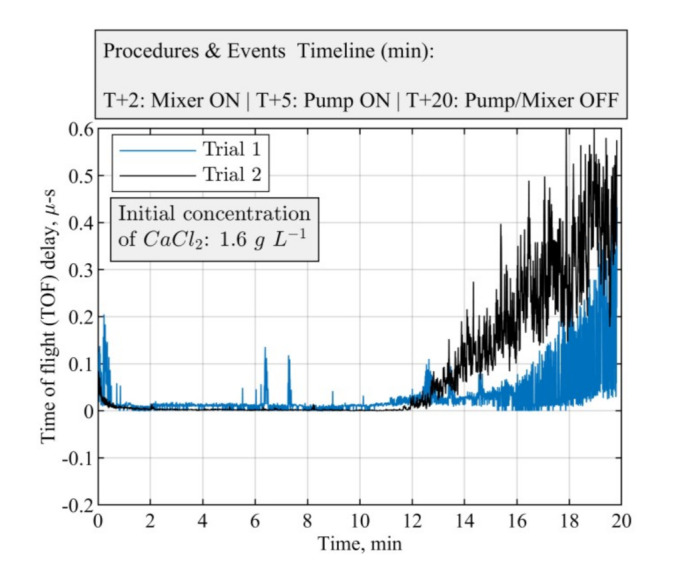
USCT measurement of the reactive crystallization process: mixing speed of 100 RPM and feed addition rate of 40 mL min^−1^. Mean value of sound speed measured for two identical crystallization experiments.

Two instances of the mean value of sound speed are presented in Figure 10. Due to the fast kinetic nature of the particulate system, the nucleation phenomena are instantaneous, which results in the formation of micron-sized particles. The mean value of sound speed obtained from averaging the ultrasound signals is not sensitive enough to react significantly to the onset of inherently stochastic nucleation, which begins at approximately T + 5 min into the process. The formation of amorphous calcium carbonate (ACC) after initiating the feed solution could be an alternative cause of the measurement delay. The formed ACC in the precipitation system will dissolve first and, then, transform within minutes to produce crystalline forms of vaterite and calcite, depending on the pH of the solution and the mixing conditions [49].

Nucleation determines the main properties of the crystal population, including the crystal polymorph, the number of crystals, and their size distribution. In the current precipitation system, there is a possibility for the following succession of mechanisms to occur [50,51]: (i) the formation and growth of ACC; (ii) the simultaneous creation of ACC surface complexation sites from which calcite starts to precipitate; (iii) the calcite growth from ACC; (iv) the creation of further calcite surface complexation sites and competitive precipitation of calcite. Hence, the observed delay between the times of 5 and 10 min in the USCT measurement could be attributed to the nature of the precipitation system. More investigations can be conducted to improve the overall operational performance of the measurement and the chemical process.

**Figure 11:**
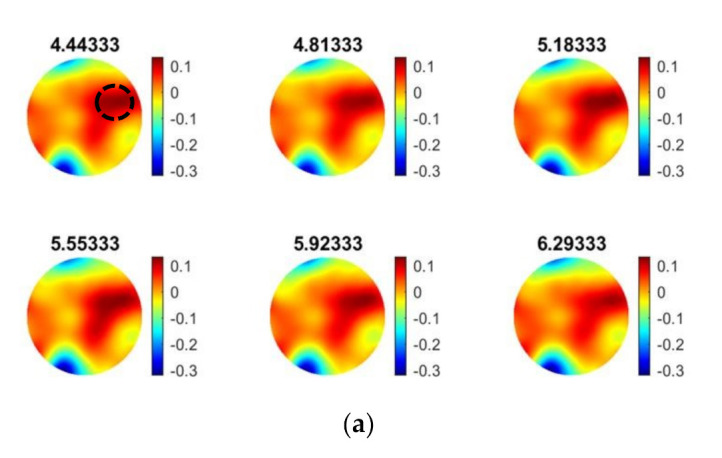

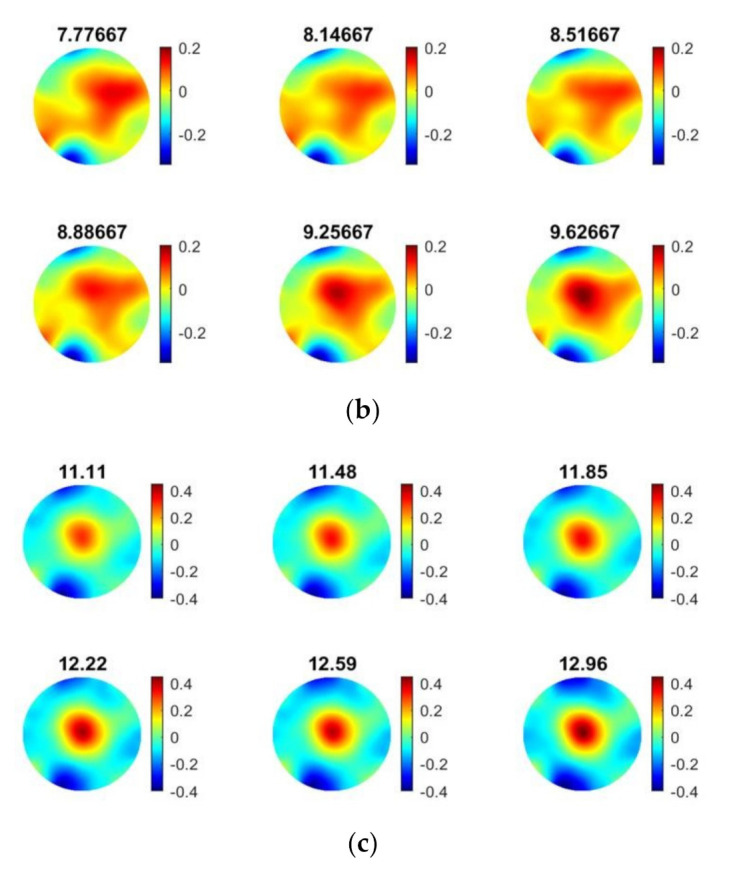
Ultrasound reconstructions during the calcium carbonate crystallization process (Trial 1 in Figure 8). Mixing speed of 100 RPM and feed addition rate of 40 ${\mathrm{mL}\;\min}^{-1}$. Parts a and b show the feeding points and c and d represent the crystal formation process. USCT reconstruction during experiments: (**a**) from T + 3.3 min to T + 6.29 min, (**b**) from T + 6.6 min to T + 9.6 min, (**c**) from T + 10 min to T + 12.9 min.

Furthermore, ultrasound tomographic reconstructions provide deeper insight into the precipitation process of calcium carbonate. Data of Trial 1 in Figure 11 were used to reconstruct the tomographic images, which shows the feeding points and the phase change throughout the process. The injection point is encircled by the black circle in the first reconstructed frame presented.

**Figure 12:**
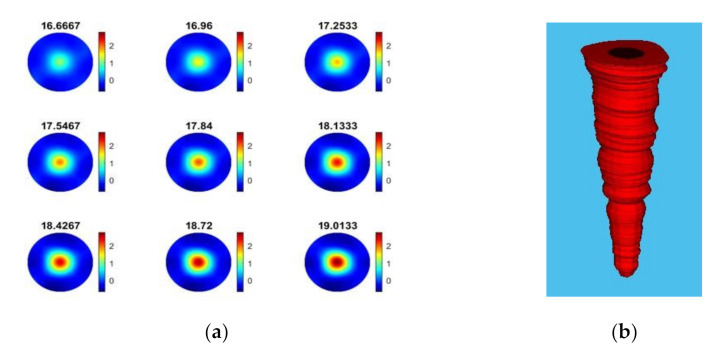
Calcium carbonate crystallization monitoring: evolution of the particle concentration and formation of a central vortex toward the end of the process (time: 16.6 min to 19 min). Experiments are related to trial 1 in Figure 10; operating parameters are listed in Table 3. (**a**) The TOF images frames, (**b**) growth of dispersed phase.

As demonstrated in the experiments with particle beads (Section 4.1), USCT measurement proved to be effective in recognizing and characterizing the bulk particle distribution in the reactor. Figure 12 shows the final stages of the calcium carbonate crystallization (based on Table 3), where the concentration of the suspension increases, and the particles are primarily accumulated in the central vortex region where a three-phase composition is formed regarding liquid solution, solid particles, and bubbles.

**Figure 13:**
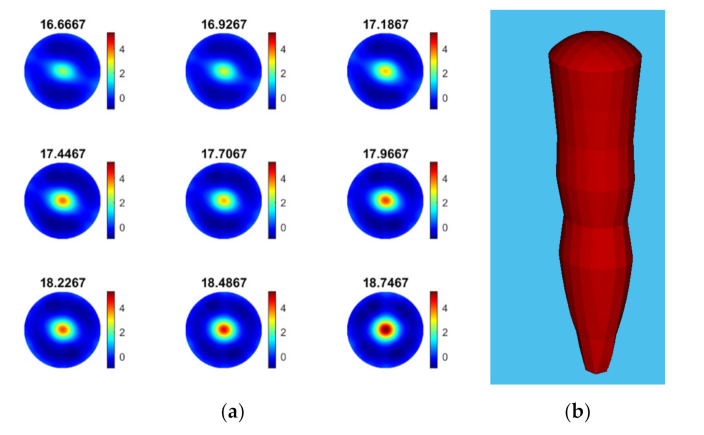
(**a**) Image reconstruction for amplitude attenuation for the same time windows as Figure 9. (**b**) Reconstructed volume depicting the growth of dispersed phase.

**Figure 14:**
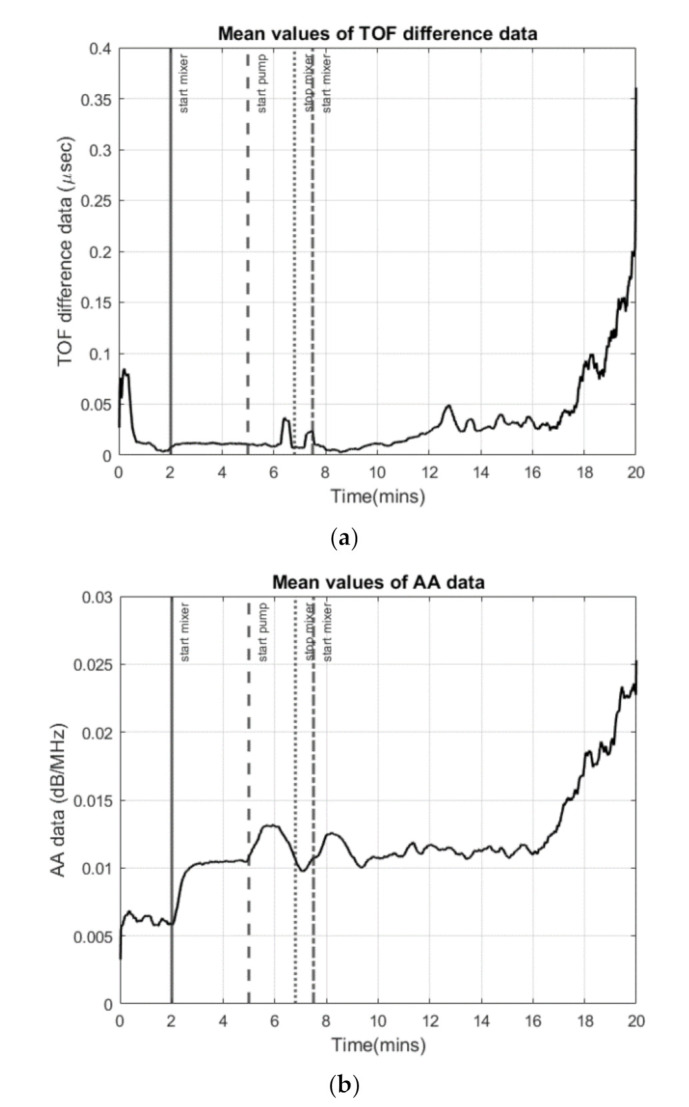
(**a**) Mean value of TOF data. (**b**) Mean value of acoustic attenuation data (in dB/MHz). In both cases: mixer on at 2 min and pump on at 5 min, stop of stirrer at 7.5 min, and stirrer starts again at 8 min.

In reactive crystallization, the time-of-flight USCT provides useful information on feeding points and the later stage of the material phase change and crystal growth. Referring to the mean value plot of TOF data in Figure 10, it is less clear to see the start and stop of the mixer and the start and stop of the pump. This may lead to some limitations for the TOF data to be used in malfunction identification (e.g., failure of the pump or mixer) and the process control implementation. In [37], we developed a full waveform USCT algorithm taking into account the acoustic attenuation, as well as the TOF data. Indeed, for the amplitude attenuation, the image reconstruction process follows a similar procedure as the transmission time-of-flight-mode USCT. Figure 13 presents AA reconstructions for the same period as Figure 12. Comparing the two figures, one can conclude that time-of-flight and acoustic attenuation provide similar results, as even the trend of increase is similar. Hence, amplitude attenuation imaging has not been presented in this work. Figure 12b shows the mean value of acoustic attenuation for a central excitation frequency of fc = 400 kHz. However, as one can see from Figure 14b below, the mean value plot for amplitude attenuation shows several clearer points of interest, such as the points where the mixer is switched on (2 min) and the pump is switched on (5 min), the stop of the stirrer (7.5 min), and the start of the stirrer (8 min). This suggests that the amplitude attenuation may provide complementary information for future control and malfunction analysis in crystallization processes. Figure 14 shows the mean of time-of-flight and amplitude attenuation data for similar time steps as Figure 10. However, plots in Figure 14 generated by smoothing the original data with a step of 10 frames. Similar imaging results can be seen between the TOF images and the AA images. Regarding TOF data, only the positive TOF delays were considered for this study as diffraction of the ultrasound is expected from the dispersed phase, especially at the low excitation frequency of 400 kHz. A multi-modality USCT, as developed in [37], and further information fusion from TOF and AA could be investigated in future studies.

To conclude, the reactive crystallization experiment was a full-scale test on live-process challenges. In the specific process, the system showed capability in detecting the injection point, as a prior localized suspension in the form of ACC. Moreover, providing real-time measurements of travel-time delays’ mean values aims at the inhomogeneities’ characterization over time and subsequently the dispersion state of the mixture. Finally, acoustic attenuation data proved to be more sensitive compared to the time-of-flight responses, as their response was significantly better in the malfunction analysis. Concluding, the mean value data for each experiment of USCT were plotted to show a global picture of the process’ progress and dynamics in all cases. Distributed sensing measurement systems such as process tomography provide the capability to measure spatiotemporal field information from within the process. Therefore, the reconstructed images aided in the injection point and in the mixture’s homogeneity characterization. The USCT images showed the feeding process, the mixing process, and material phase changes during the crystallization process. Moreover, USCT data were further analyzed to estimate the settling velocity and settling time of particles in the suspension.

## 5. Conclusions

Transmission-mode USCT was examined for industrial process monitoring. The experiments were designed in such a way that enables critical evaluation of the USCT and its usability for process monitoring and potentially for process control. For transmission USCT, we implemented both time-of-flight, allowing speed-of-sound imaging, and amplitude attenuation imaging. Although we mostly showed the time-of-flight data, the amplitude attenuation was also examined and provided similar or, in some cases, complementary information on the state of the process. The major events on the process, such as feeding, and switching on and off the pumps and the stirrer, could be seen in the data. These indicators could be integrated into the process and be used for process malfunction. CO_2_ capturing via reactive crystallization is becoming a new tool to reduce the negative environmental impact of CO_2_, helping in net-zero targets. There is clear indication that the USCT tool can be used to monitor such a process noninvasively and could then become a practical tool to process design that can maximize the overall process yield contributing to affordable carbon capture and carbon reduction. To develop a complete understanding of a complex industrial process, such as carbon capture experiments, it is likely that a multi-modality sensing and imaging approach will be required. In such a multimodality imaging setup, the data-rich USCT is likely to play a vital role. The work of this paper demonstrates that USCT is a very attractive and proven tool to many industrial applications. Hopefully, this work will stimulate further commercial exploitation of the USCT technology.

## Figures and Tables

**Table 1:** The main design features, structural parameters, and values of the utilized ultrasound tomography system.

Parameter	Value
Transducers’ frequency	400 kHz
Number of pulses	6
Supply voltage	+72/−72 V
Pull to the ground after extortion	45.2 dB
Gain the first stage	1 *v/v*
Analog filter	Band-pass 350 kHz
Convert to envelope	HI
Offset-RAW signal	1.75 V
Offset-ENVELOPE signal	0 V
Number of channels	16

**Table 2:** Particle beads detection experimental procedure.

Steps	Task
1	T + 0 min: Reactor filled with 3 L of water
2	T + 0 min: USCT measurements start
3	T + 1 min: Addition of 100 g of particle beads at 1 min
4	T + 2 min: Mixer starts at 200 RPM at 2 min
5	T + 3 min: Stop mixer at 3 min
6	T + 4 min: End of USCT measurements at 4 min

**Table 3:** Main operating parameters for the CaCO_3_ crystallization experiments.

Parameter	Unit	Value
${\mathrm{CaCl}}_{2}$ concentrations	${g\;L}^{-1}$	0, 1.6
NaOH concentration at the feed	${\mathrm{mol}\;L}^{-1}$	12.1 ± 0.1
${\mathrm{CO}}_{3_{\left(\mathrm{aq}\right)}}^{2-}$ concentration at the feed	${\mathrm{mol}\;L}^{-1}$	0.15 ± 0.5
Feed addition rate	mL min^−1^	40
Impeller diameter	m	0.07
Stirring rate	rpm	100
Impeller tip speed	${m\;s}^{-1}$	0.37

## Data Availability

Not applicable.
